# A general lithography-free method of microscale/nanoscale fabrication and patterning on Si and Ge surfaces

**DOI:** 10.1186/1556-276X-7-110

**Published:** 2012-02-08

**Authors:** Huatao Wang, Tom Wu

**Affiliations:** 1 Division of Physics and Applied Physics, School of Physical and Mathematical Sciences, Nanyang Technological University, Singapore, 637371, Singapore; 2 School of Materials Science and Engineering, Harbin Institute of Technology in Weihai, Weihai, 264209, China

**Keywords:** lithography-free patterning, nanostructures, pits, silicides, germanides

## Abstract

Here, we introduce and give an overview of a general lithography-free method to fabricate silicide and germanide micro-/nanostructures on Si and Ge surfaces through metal-vapor-initiated endoepitaxial growth. Excellent controls on shape and orientation are achieved by adjusting the substrate orientation and growth parameters. Furthermore, micro-/nanoscale pits with controlled morphologies can also be successfully fabricated on Si and Ge surfaces by taking advantage of the sublimation of silicides/germanides. The aim of this brief report is to illustrate the concept of lithography-free synthesis and patterning on surfaces of elemental semiconductors, and the differences and the challenges associated with the Si and the Ge surfaces will be discussed. Our results suggest that this low-cost bottom-up approach is promising for applications in functional nanodevices.

## Introduction

In the semiconductor industry, lithography is indispensable to achieve desired microscale and nanoscale patterns on semiconductor surfaces [1,2]. Lithography methods in various forms are also widely used in many research fields for making functional devices. As a vital part of interconnects, metal silicides have considerable uses in modern integrated circuits, and their patterning attracts lots of interests [3]. In the conventional top-down lithography technology, expensive equipment with predesigned masks and collimated light sources are employed, which involve complex and time-consuming processing steps. Electron beam lithography writes nanoscale patterns without the use of any physical mask, but its high equipment cost and low patterning speed stifle its widespread applications [4]. In recent years, there has been intensified interest on developing bottom-up techniques of lithography-free patterning. Techniques like laser interference and nanosphere lithography are alternative routes toward achieving regular nanoscale patterns without using any mask [5-10]. Recently, Wu et al. reported a novel lithography-free method of forming nanopores in plastic membranes using laser heating [11]. Feng et al. developed a novel process to fabricate submicron-scale silicon [Si] pillars, which are promising as catalyst support structures in fuel cell applications [12].

Germanium [Ge] differs from Si in that the supply for Ge is limited by the availability of exploitable sources, while the supply of Si is only limited by the production capacity. As a result, Si dominates the microelectronic industry and accounts for the construction of most devices, but it does not operate at frequencies above a few gigahertzes. On the other hand, Ge as another notable group IV element has a smaller bandgap and a much higher mobility. In general, compared with Si, the fabrication and patterning of Ge surfaces have been much less investigated, and there are few examples of lithography-free patterning of Ge surfaces in literature.

Here, we demonstrate a vapor transport-based method to fabricate shape-controlled nanostructures and pits on both Si and Ge surfaces. This facile method takes advantage of the anisotropic diffusion/reaction of metals in crystalline elemental semiconductors and the ensuing morphology-controlled growth or sublimation of silicides/germanides. Our results suggest that this method can serve as a general lithography-free approach to fabricate metal-semiconductor compounds and make micro-/nanoscale patterns on semiconductor surfaces. In this brief report, we will not exhaust all the details related to the complex nanoscale reactions between metals and semiconductors; instead, we will use some examples to illustrate the concept and elucidate some general considerations in this synthesis/patterning strategy.

## Methods

### Fabrication of morphology-controlled nanoscale silicides/germanides and microscale/nanoscale pits on Si and Ge surfaces

Our experiments were carried out in a home-built vapor transport growth system that comprises a quartz tube heated by a horizontal tube furnace (Lindberg Blue Mini-Mite, Thermo Fisher Scientific, Waltham, MA, USA). Si and Ge wafers with <111>, <110>, and <100> orientations were cut into small pieces with a typical size of 6 × 6 mm and cleaned by ultrasonication in ethanol. In most cases, the native oxide layer on substrates was not etched off because the metal vapor can 'penetrate' the oxide and reach the semiconductor underneath. To obtain a uniform distribution of pits, the substrates were pretreated with oxygen plasma, and Au nanoparticles [NPs] with a size of 30 to 40 nm were dispersed on the substrate surfaces. Metal chloride or mixture powder of metal oxide and graphite was used as the source for metal vapors, e.g., iron chloride (99.99%; Sigma-Aldrich Corporation, St. Louis, MO, USA) was used as iron source, a mixed powder of copper oxide (99.99%, Sigma-Aldrich) and graphite (Riedel-de Haën AG, Buchs, St. Gallen, Switzerland) with a weight ratio of 1:1 was used as the copper source, and a mixed powder of cobalt oxide (99.99%, Sigma-Aldrich) and graphite (Riedel-de Haën) with a weight ratio of 1:1 was used as the cobalt source.

In the furnace tube, the substrates were placed 3 to 10 cm downstream from the source powder. During the synthesis, Ar was introduced as the carrying gas with a constant flow rate of 50 to 100 sccm, and the pressure inside the quartz tube was maintained at 1 to 20 mbar. To achieve reproducible synthesis, the source and the substrate temperatures in the range of 700°C to 1,000°C were carefully calibrated. The furnace temperature plays a crucial role in the lithography-free patterning. The furnace tube was heated for a predetermined period of time and then quickly cooled down to room temperature.

### Characterizations

A JEOL JSM-6700F field emission scanning electron microscope [SEM] (JEOL Ltd., Akishima, Tokyo, Japan) was used to study the sample morphology. The crystal structure and composition were determined by X-ray diffraction (Bruker AXS D8 Advanced powder diffractometer with CuKα radiation; BRUKER OPTIK GMBH, Ettlingen, Germany) and energy dispersive X-ray spectrometry.

## Results and discussion

### Patterning process and mechanism

**Figure 1:**
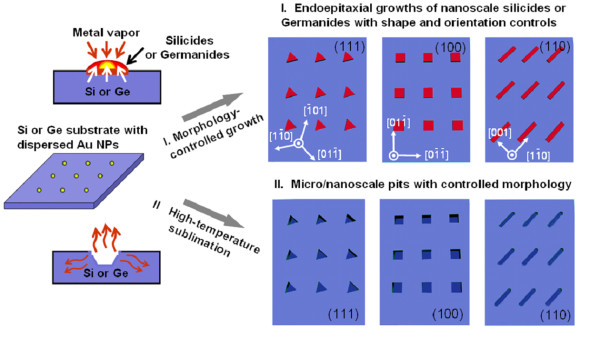
**Schematic illustrating the lithography-free micro-/nanoscale patterning on Si and Ge surfaces**. I. Endoepitaxial growth of nanoscale silicides or germanides with shape and orientation controls. Either triangle-, square-, or wirelike silicides/germanides can be synthesized, depending on the orientation of the substrates used. II. Morphology-controlled micro-/nanoscale pits via tailored sublimation. Such inverted structures on Si/Ge surfaces can be achieved if the furnace temperature is high enough to induce the sublimation of the metal-semiconductor compounds. In both processes, Au NPs are used to trap metal vapors at high temperature, which effectively guides the locations of reactions.

Figure 1 shows the general scheme of achieving lithography-free nanoscale synthesis and patterning on Si and Ge surfaces. Uniform Au NPs with sizes of 30 to 40 nm were dispersed on the substrates, and their liquid-state surfaces at the high synthesis temperatures help to absorb the metal vapors to initiate the localized nanoscale reactions. The growth usually takes place for 5 to 30 min, and the reactions can be well controlled by adjusting the growth parameters.

As illustrated in part I of Figure 1, nanostructures of silicides/germanides can endoepitaxially grow into Si and Ge surfaces. The semiconductor substrates not only serve as the supporting media, but also actively participate in the growth of nanostructures by providing the Si or Ge atoms. In this sense, the synthesis is quite different from the conventional epitaxial growth where the substrates only serve as the templates and do not participate in the reactions. The shape control of the silicide/germanide nanostructures was realized by selecting the orientation of substrates. As reported previously [13-15], nanostructures and pits in the shapes of triangle, square, and wire could be achieved on substrates with the orientation of <111>, <100>, and <110>, respectively. This formation of well-defined shapes can be explained by the anisotropic diffusion of metal species and the associated anisotropic reaction rates in elemental semiconductors.

It should be noted that the synthesis temperature must be carefully tailored: On the one hand, the growth cannot take place if the temperature is too low, but on the other hand, the shape control would be compromised if the temperature is too high. In the complex ternary phase diagrams of metal-semiconductor-oxygen [16], the various Gibbs energies determine the stable compounds and the tie lines at fixed temperatures and pressures. Thus, the thermodynamics of reactions must be carefully considered in order to achieve the desired nanostructures of silicides/germanides. Furthermore, there is always a competition between the 'in-plane' endoepitaxial growth and the 'out-of-plane' nanowire growth [17,18], which involve complex growth dynamics, determined not only by the metal species used, but also by the detailed synthesis conditions.

Here, we take the shape-controlled synthesis of copper silicide nanostructures as an example to illustrate the mechanism. According to the Au-Si binary phase diagram, at temperatures as low as 300°C to 400°C, Au NPs already start forming Au-Si eutectic alloys with the Si substrate. As the synthesis temperature rises beyond approximately 600°C, copper oxide in the source powder is reduced by graphite to generate Cu vapor, which is then absorbed by the Au-Si alloy NPs to form Au-Si-Cu alloy nanoparticles. As the substrate temperature rises higher, Cu_3_Si gradually precipitates from the alloy NPs and endoepitaxially grows on the Si surface. The growth of the Cu_3_Si nanostructures slows down as their size becomes larger because the Cu vapor must diffuse through the formed silicide to continue the reaction with Si, and uniform sizes can be achieved by controlling the growth time. We should note that Cu_3_Si nanostructures can still be grown even without the Au NPs, which may be a result of the preferred trapping and absorption of Cu vapor at some defective features on the Si surface, but the density and size of the Cu_3_Si nanostructures are less controlled.

Interestingly, formation of 'hollow' micro-/nanoscale pits can also be achieved if the metal silicides/germanides have low sublimation temperatures, and the mechanism is illustrated in part II of Figure 1. This transition from solid nanomaterials to hollow pits can be accelerated by increasing the furnace temperature and/or lowering the reaction pressure. We can use the formation of micro-/nanoscale pits on Si substrates assisted by cobalt vapor as an example to reveal the mechanism of this lithography-free patterning [14]. Similar to the Cu_3_Si case, nanostructures of cobalt silicide can form on Si surface, but CoSi_2 _is not stable as Cu_3_Si temperatures are above 860°C and ambient pressure is 1 to 20 mbar, and the sublimation of CoSi_2 _effectively erodes the reactive area to form the pits. It should be noted that the sublimation can happen at lower temperatures than the bulk counterparts due to the remarkable size effect in nanostructures. In addition, CoSi_2 _is prone to decompose at temperatures higher than its thermal stability temperature of 900°C. In the synthesis, Si is continuously consumed by reacting with Co, and the well-defined shapes are results of the anisotropic reaction and sublimation which depend on the orientation of the substrates.

The use of metals in etching nanostructures on semiconductor surfaces is analogous to the recently developed technique of metal-assisted chemical etching [MACE] [19,20]. In MACE, Si nanowires can be obtained by depositing metals (Ag or Pt) on Si and by subsequent etching. However, in our case, HF etching was not used, and the vapor-based reaction mechanism is different.

The above-revealed mechanism of forming nanostructures and pits applies to both Si and Ge as they belong to the same group IV elements and have similar chemical properties. Both Si and Ge crystallize in a diamond cubic crystal structure, and their lattice mismatch is about 4%. Also, their heat of vaporization is similar: 359 and 334 kJ·mol^-1 ^for Si and Ge, respectively. Processing temperature and pressure have determining effects on both the growth and the sublimation of silicides/germanides; therefore, they must be carefully controlled. It is very important to note that the chemical properties of Si and Ge surfaces are different, so the reaction conditions must be tailored individually. Unlike Si whose surface in air is readily covered by oxide, elemental Ge oxidizes slowly at 250°C. Furthermore, Si has a high melting point of approximately 1,400°C, while Ge melts at a much lower temperature of 938°C. Although the surface of a particular material often melts at a lower temperature than the bulk, we can expect that melting of the Ge surface is more readily to occur than that of the Si surface. Indeed, in general, we found that Ge appears to be more volatile, and the micro-/nanoscale reactions on the Ge surface are more difficult to control. For example, copper silicide can form regular nanostructures on Si surfaces, but our attempt to form copper germanide on Ge surfaces was not successful although extensive search of the appropriate growth windows was conducted. Instead, iron germanide gave much better results in terms of synthesis controls, and the detailed mechanisms behind these element-dependent reactions remain largely unknown. We also found that the sublimation temperatures of silicides and germanides are quite different even when the same metal is used; therefore, as illustrated, we used the reactions with different metals to illustrate the patterning strategies.

### Endoepitaxial growth of silicide and germanide nanostructures with shape and orientation controls

**Figure 2:**
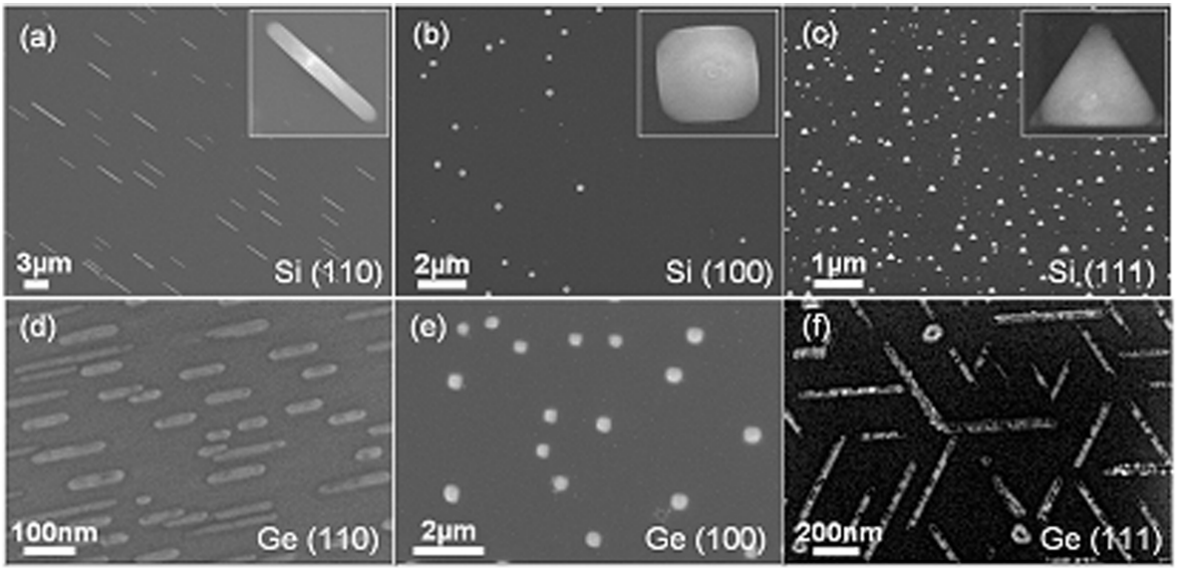
**Endoepitaxial growth of silicide or germanide nanostructures with shape and orientation controls**. The upper row shows the SEM images of wire-, square- and triangle-like copper silicide nanostructures grown on (**a**) Si(110), (**b**) Si(100), and (**c**) Si(111) substrates, respectively. Insets show the magnified views of individual nanostructures. The lower row shows the SEM images of shape-controlled iron germanide nanostructures grown on (**d**) Ge(110), (**e**) Ge(100), and (**f**) Ge(111) substrates, respectively.

Figure 2a, b, c shows representative SEM images of copper silicide nanostructures on Si surface. The substrate orientation and symmetry dictate the shape of the nanostructures. Nanoscale wires, squares, and triangles were observed on Si(110), Si(100), and Si(111) substrates, respectively. Nanowires have widths approximately between 200 and 400 nm, and lengths approximately between 0.6 and 6.0 μm. The equilateral nanosquares and nanotriangles exhibit lateral dimensions approximately from 100 to 400 nm. In a given sample, all the nanostructures have the same orientation. For nanowires, the growth direction was determined to be along the Si $<1\bar{1}0>$ direction. For nanosquares, the four edges are along Si <011>, $<01\bar{1}>$, $<0\bar{1}\bar{1}>$, and $<0\bar{1}1>$, respectively. For nanotriangles, the three edges are along Si $<1\bar{1}0>$, $<01\bar{1}>$, and $<\bar{1}01>$, respectively.

Germanide nanostructures with shape controls can be grown on Ge surfaces. However, we found that the control on the synthesis of copper germanide nanostructures is quite poor, so here, we used iron germanide as an example instead. Iron chloride was used to release the iron vapor at the growth temperature of 850°C. Figure 2d, e shows that germanide nanostructures with shapes of wire and square are endoepitaxially grown on Ge(110) and Ge(100) substrates. Moreover, wirelike nanostructures with three directions separated by 120° were found on Ge(111) (Figure 2f), which is different from the triangle-type silicide nanostructures and can be a result of different growth kinetics.

### Microscale/nanoscale pits with controlled morphology on Si and Ge surfaces

**Figure 3:**
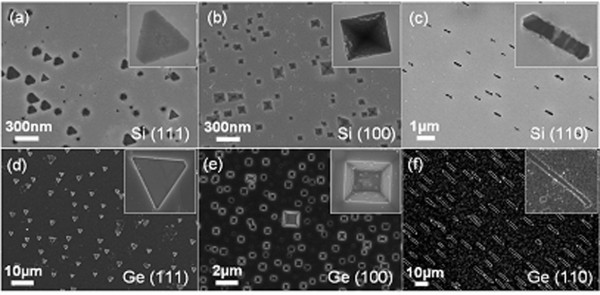
**Micro-/nanoscale pits with controlled morphologies on Si and Ge surfaces**. The upper row shows the SEM images of triangle-, square- and wirelike pits formed on (**a**) Si(111), (**b**) Si(100), and (**c**) Si(110) surfaces, respectively. SEM images of triangle-, square- and wirelike pits formed on (**d**) Ge(111), (**e**) Ge(100), and (**f**) Ge(110), respectively. Insets show magnified views of individual pits.

Figure 3a, b, c shows representative SEM images of regular pits formed on Si surfaces using the mixed powder of cobalt oxide and graphite as the metal source. We should note here that in general, the synthesis temperature was intentionally set much higher than what is usually used for silicide growth. It was found that the substrate orientation and symmetry reproducibly dictate the shape of the 'inverted' nanostructures. Nanopits in shapes of triangle, square, and wire were observed on Si(111), Si(100), and Si(110) substrates, respectively, and all the nanopits have the same orientation in a given sample. The equilateral square and triangle pits exhibit lateral dimensions approximately from 50 to 250 nm, and approximately from 60 to 250 nm, respectively. The wire-shaped pits have widths approximately between 100 and 200 nm, and lengths approximately between 400 and 1,300 nm. In general, by carefully adjusting the reaction temperature and duration, we can reproducibly control the dimension of the pits from under 70 nm to several micrometers.

By examining their angles relative to the substrate coordinates, the pit edges and exposed surfaces can be identified. For triangle pits, the three edges are along Si $<1\bar{1}0>$, $<01\bar{1}>$, and $<\bar{1}01>$. For square pits, the four edges formed on Si(100) substrate are along Si <011>, $<01\bar{1}>$, $<0\bar{1}\bar{1}>$, and $<0\bar{1}1>$. For wire-shaped pits, the growth direction is Si $<1\bar{1}0>$ with the width direction of <001>. The inset images in Figure 3a, b, c show magnified views of individual pits. Generally, Si {111} is the most frequent exposed plane, which has the minimum surface energy among all the crystalline planes in Si [21,22]. For square and triangle nanopits, all the exposed side planes belong to {111}; for wire-shaped nanopits, the broad sidewalls also belong to {111}.

Figure 3d, e, f shows the SEM images of regular pits grown on Ge surface with the mixed powder of copper oxide and graphite as the source for producing copper vapor. This is a good example to illustrate the differences between the reaction chemistry of the Si and Ge surfaces. In the case of Si, nanostructures of copper silicides can be routinely achieved, but in the case of Ge, we only observed the formation of pits, which can be a result of the much lower sublimation temperature of copper germanide. The insets in Figure 3d, e, f show the magnified images of individual pits. Nanopits in shapes of triangle, square, and wire were observed on Ge(111), Ge(100), and Ge(110) substrates, respectively, and all the nanopits have the same orientation in a given sample. The equilateral triangle and square pits exhibit lateral dimensions approximately from 1 to 2 μm, and approximately from 500 nm to 2 μm, respectively. The wire-shaped pits have widths approximately between 2 and 3 μm, and lengths approximately between 7 and 15 μm. In addition, the orientations of the pits on Ge are consistent with the ones on Si, which give further support to the formation mechanism, but in general, we found that the Ge surfaces are much more volatile than the Si surfaces, and the control of patterning on the Ge surfaces is quite challenging, which clearly warrants further investigations.

## Conclusions

In this work, lithography-free growth/patterning on Si and Ge surfaces has been realized by controlling the reactions between substrates and metal vapors. Au NPs can effectively absorb the metal vapors at high temperature, initiating the micro-/nanoscale reactions of silicides and germanides and giving rise to nanostructures with well-defined morphology and orientation. Furthermore, with tailored sublimation, metal vapors can 'etch' the semiconductor surfaces, giving rise to pitlike structures with well-controlled morphology and orientation.

This versatile approach is cost effective and convenient although the position control is not as accurate as the conventional lithography-based techniques. There remain many challenges before such a lithography-free technique can be widely employed in making patterns on elemental, or even compound, semiconductors. The reactions between metal vapors and semiconductors are quite complex, and the synthesis windows for individual combinations can be significantly different. Therefore, lots of future efforts are needed to examine and document these nanoscale reactions on semiconductor surfaces, which is clearly beyond the scope of this paper. The process temperatures for initiating these reactions in vapor transport are quite high, which should be reduced in order to be compatible with the existing device technologies. Overall, the endoepitaxial growth of regular micro-/nanoscale structures and the formation of well-defined pits on Si and Ge surfaces offer excellent morphology controls and may find uses in nanoelectronic devices.

## Abbreviations

NPs: nanoparticles; SEM: scanning electron microscope.

## Competing interests

The authors declare that they have no competing interests.

## Authors' contributions

TW conceived the idea and designed the experiments. HW carried out the experiments and data analysis. Both authors drafted the manuscript and approved the final version.
